# Utility of plasma cell‐free DNA in metastatic castration‐resistant prostate cancer

**DOI:** 10.1002/iju5.12172

**Published:** 2020-06-07

**Authors:** Itsuto Hamano, Shingo Hatakeyama, Tomoko Hamaya, Kyo Togashi, Teppei Okamoto, Hayato Yamamoto, Tohru Yoneyama, Takahiro Yoneyama, Yasuhiro Hashimoto, Chikara Ohyama

**Affiliations:** ^1^ Department of Urology Hirosaki University Graduate School of Medicine Hirosaki Japan; ^2^ Department of Advanced Blood Purification Therapy Hirosaki University Graduate School of Medicine Hirosaki Japan; ^3^ Department of Advanced Transplant and Regenerative Medicine Hirosaki University Graduate School of Medicine Hirosaki Japan

**Keywords:** androgen receptor, biomarker, castration‐resistant prostate cancer, cell‐free DNA, concentration

## Abstract

**Introduction:**

Cell‐free DNA is suggested as a prognostic biomarker in metastatic castration‐resistant prostate cancer. However, it remains unknown which parameter of cell‐free DNA is correlated with the progression and prognosis of metastatic castration‐resistant prostate cancer.

**Case presentation:**

A 75‐year‐old man with newly diagnosed prostate cancer (serum prostate‐specific antigen 4891 ng/mL, Gleason score 4 + 5 = 9, cT3bN1M1) was referred to our department. He first received sequential hormonal therapies and was consequently diagnosed metastatic castration‐resistant prostate cancer 64 months after initial treatment. He underwent serial examinations of plasma cell‐free DNA, including concentration, androgen receptor amplification, TP53 point mutation, and PTEN loss. Only the cell‐free DNA concentration increased along with disease progression and declined after the administration of abiraterone and enzalutamide.

**Conclusion:**

This case presented that cell‐free DNA concentration was possibly correlated with response to castration‐resistant prostate cancer treatment and disease progression. Cell‐free DNA concentration was proposed as a potential prognostic biomarker of metastatic castration‐resistant prostate cancer.

**Abbreviations & Acronyms**
ALP: alkaline phosphatase
AR‐amp: androgen receptor amplification
ARAT: androgen receptor‐axis‐targeted therapy
CAB: combined androgen blockade
cfDNA: cell‐free DNA
CNV: copy number variation
CT: computed tomography
LDH: lactate dehydrogenase
mCRPC: metastatic castration‐resistant prostate cancer
PCa: prostate cancer
PSA: prostate‐specific antigen

**Keynote message**

We present a case of a patient with mCRPC who underwent serial examinations of plasma cfDNA. His cfDNA concentration potentially indicated disease progression and response to treatment with abiraterone and enzalutamide. Further studies are necessary to identify predictive parameters of cfDNA for the progression and prognosis of mCRPC.

## Introduction

mCRPC remains a major cause of male cancer deaths.^1^, ^2^, ^3^, ^4^, ^5^ Not all patients with mCRPC present remarkable increases in serum PSA level as the disease progresses, and presently there are no useful biomarkers that predict disease progression of mCRPC.^6^, ^7^, ^8^, ^9^, ^10^ Recently, the clinical implication of cfDNA in mCRPC has been studied.^11^, ^12^ However, it remains unknown which parameter of cfDNA is correlated with the progression and prognosis of mCRPC.^13^ We have examined longitudinal changes in various parameters of cfDNA in patients with mCRPC at our hospital. We present a case suggesting that the plasma cfDNA concentration potentially indicated mCRPC progression and response to treatment with abiraterone and enzalutamide.

## Case presentation

**Fig. 1:**
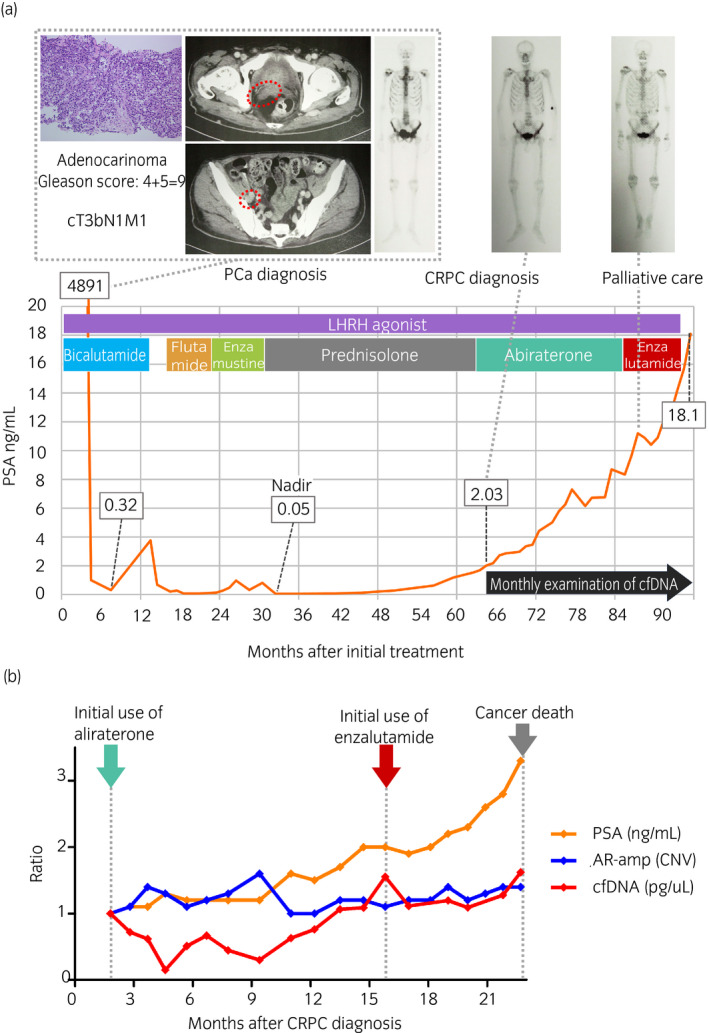
Treatment sequence and outcomes in a 75‐year‐old man with mCRPC (a) and longitudinal changes in plasma cfDNA concentration and other parameters. In this case, metastatic hormone‐sensitive PCa (cT3N1M1) progressed to mCRPC in 64 months. Red dotted circles in the CT images indicate PCa invasion to seminal vesicle and metastasis to the right external iliac lymph nodes. Although the image findings with bone scintigraphy did not present disease progression, his general conditions gradually became worse until cancer death 30 months after the CRPC diagnosis (a). The graph shows longitudinal changes in the ratios of PSA, cfDNA concentration, and AR‐amp in cfDNA compared with baseline. cfDNA concentration (red line) was the only parameter that declined after administration of abiraterone and enzalutamide and presented the increase along with disease progression of mCRPC (b).

A 75‐year‐old man with PCa (serum PSA 4891 ng/mL, Gleason score 4 + 5 = 9, cT3bN1M1, Fig. 1a, upper left panel) was referred to our department. He was first treated with CAB by leuprorelin and bicalutamide and later received sequential hormonal therapies. He was diagnosed with mCRPC with a PSA of 2.03 ng/mL 64 months after the initial CAB (Fig. 1a, lower panel). We started the monthly examination of cfDNA to monitor the longitudinal changes in plasma cfDNA concentration (pg/mL), CNV of AR‐amp, TP53 point mutation (p.R175H, %), and PTEN loss (p.R233*, %) as recently suggested mCRPC biomarkers in cfDNA.^12^ In addition, the reproducibility of cfDNA concentration was confirmed as the error range was within 10% by multiple (two or more) measurements.

**Fig. 2:**
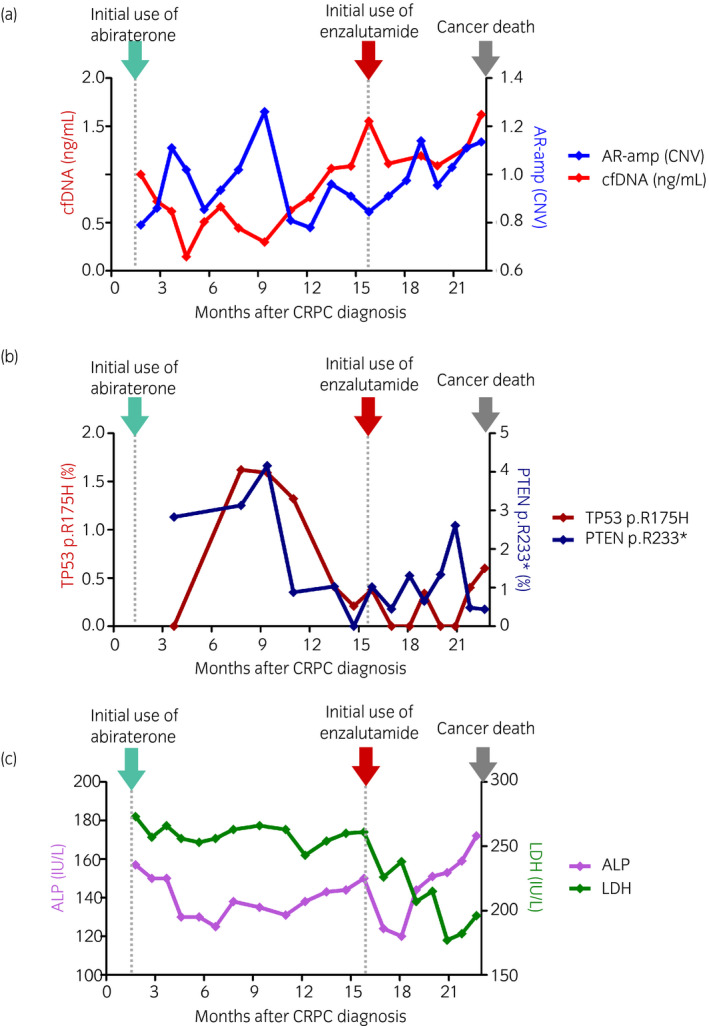
Longitudinal changes in the absolute value of potential biomarkers. While plasma cDNA concentration implied a reaction to the use of abiraterone and enzalutamide, AR‐amp in cfDNA did not show a remarkable association with ARAT treatment (a). Frequency of TP53 point mutation and PTEN loss appeared to decline at 9 months after the administration of abiraterone (b). ALP and LDH declined after administration of abiraterone and enzalutamide ©.

Although we administered abiraterone acetate (1000 mg/day) followed by enzalutamide (160 mg/day), PSA continuously rose during treatment. We could not detect radiologic progression by CT or bone scintigraphy (Fig. 1a, upper right panels) until his death. His main symptom was fatigue, which did not progress until 1 year after the administration of abiraterone. After that term, his general condition became worse with the PSA rose until he died 94 months since the PCa diagnosis. The cause of death was considered PCa progression due to no other findings for the cause of death. Meanwhile, AR‐amp was not correlated with the PSA rise (Figs 1b,2a), and frequency of TP53 point mutation and PTEN loss appeared to decline at 9 months after the administration of abiraterone (Fig. 2b). cfDNA concentration and serum ALP and LDH demonstrated responses to the administration of abiraterone and enzalutamide, which was not detected by serum PSA value (Figs 1b,2a,c).

## Discussion

This case implied that plasma cfDNA concentration is a potential biomarker for both progression of mCRPC and response to ARAT. There has been an unmet need for a noninvasive procedure that can examine cancer cells other than tissue biopsies. Thus, cfDNA has been focused on as a potential biomarker that demonstrates gene information as well as the total amount of apoptotic and/or necrotic cells in the entire body.^12^ However, it remains unknown which cfDNA parameters might precisely predict prognosis and response to treatment in mCRPC.

While the association between total cfDNA amount and cancer prognosis has been gradually recognized in colorectal,^13^ lung,^14^ and breast cancers,^15^ only a few studies have proposed the clinical implication of total cfDNA in PCa.^16^, ^17^ Kienel *et al*. assessed cfDNA before taxane‐based chemotherapy in 59 patients with CRPC and concluded that cfDNA concentration before therapy was an independent predictor of overall survival.^16^ Also, Mehra *et al*. investigated 571 patients with mCRPC and suggested that cfDNA concentration before taxane‐based chemotherapy was a predictive factor for shorter radiologic progression‐free survival and overall survival.^17^ However, former studies have not investigated the longitudinal change in cfDNA concentration along with CRPC treatment. In the present case, cfDNA concentration declined but PSA did not remarkably change after each administration of ARAT, which implied that cfDNA is potentially a more sensitive biomarker for tumor burden than PSA in mCRPC.

Because of the heterogeneity of mCRPC and the sequence of molecular events in disease progression and therapy resistance,^18^ it remains challenging to utilize specific DNA alterations in cfDNA as a biomarker for mCRPC. Among various DNA alterations, AR gene status in cfDNA has been reported as a factor associated with therapy resistance in CRPC.^18^, ^19^ Conteduca *et al*. analyzed AR copy number and mutations in 73 chemotherapy‐naïve and 98 post‐docetaxel patient groups and identified a correlation between AR gain and worse OS in both groups.^18^ Also, Sumiyoshi *et al*. analyzed the AR status of 102 patients with CRPC and suggested the AR aberrations, especially AR‐amp, in pretreatment cfDNA were associated with poor response to abiraterone.^19^ However, AR aberrations in cfDNA are not always detected in patients with CRPC.^11^, ^18^, ^19^ The longitudinal observation in our case suggested inconsistent changes in AR‐amp along with CRPC progression.

Although aberrations in TP53 and PTEN are potential biomarkers of CRPC,^12^ single‐point mutation could not predict disease progression because of the wide variety of mutations in those oncogenes. Indeed, we only analyzed specific point mutations of TP53 and PTEN, which did not imply correlation with mCRPC progression. Meanwhile, the frequency of TP53 and PTEN aberrations appeared to decline after the use of ARAT in this case. As no previous studies have reported such phenomenon, this case may be one of the first studies that suggested the clinical implication of longitudinal change in TP53 and PTEN aberrations after CRPC treatment.

This case implied that cfDNA concentration is a potential important correlate in understanding whole‐body conditions in patients with end‐stage mCRPC. Interestingly, the patient’s symptom and performance status did not become worse during the cfDNA concentration declined after the administration of abiraterone. Less invasive liquid markers may be beneficial for predicting the prognosis of patients with CRPC in a terminal state. This case also presented a trend in cfDNA concentration similar to those seen in conventional serum markers such as LDH and ALP. Moreover, a recent study suggested that aberrant serum N‐glycans are associated with disease progression and poor prognosis in patients with mCRPC.^20^ Further studies are warranted to clarify whether cfDNA concentration can serve as a biomarker in end‐stage mCRPC.

In conclusion, our case report of a patient with mCRPC proposed that plasma cfDNA concentration was possibly correlated with response to ARAT and disease progression not precisely demonstrated by PSA, AR‐amp, or imaging modalities. Future studies need to investigate cfDNA as a potential prognostic biomarker of mCRPC.

## Ethical consideration

The genetic tests for this research project have been approved by the Ethics Committee of Hirosaki University Hospital (Approval No. 2019‐094). Written informed consent was obtained from the subject.

## Conflict of interest

The authors declare no conflict of interest.
